# Phenotypic and metabolic traits of commercial *Saccharomyces cerevisiae* yeasts

**DOI:** 10.1186/s13568-014-0039-6

**Published:** 2014-05-10

**Authors:** Catarina Barbosa, Patrícia Lage, Alice Vilela, Arlete Mendes-Faia, Ana Mendes-Ferreira

**Affiliations:** 1 Institute for Biotechnology and Bioengeneering – Centre of Genomics and Biotechnology, Universidade de Trás-os-Montes e Alto Douro, Vila Real, Portugal

**Keywords:** Yeast, Nitrogen requirements, Stress resistance, Alcoholic fermentation

## Abstract

Currently, pursuing yeast strains that display both a high potential fitness for alcoholic fermentation and a favorable impact on quality is a major goal in the alcoholic beverage industry. This considerable industrial interest has led to many studies characterizing the phenotypic and metabolic traits of commercial yeast populations. In this study, 20 *Saccharomyces cerevisiae* strains from different geographical origins exhibited high phenotypic diversity when their response to nine biotechnologically relevant conditions was examined. Next, the fermentation fitness and metabolic traits of eight selected strains with a unique phenotypic profile were evaluated in a high-sugar synthetic medium under two nitrogen regimes. Although the strains exhibited significant differences in nitrogen requirements and utilization rates, a direct relationship between nitrogen consumption, specific growth rate, cell biomass, cell viability, acetic acid and glycerol formation was only observed under high-nitrogen conditions. In contrast, the strains produced more succinic acid under the low-nitrogen regime, and a direct relationship with the final cell biomass was established. Glucose and fructose utilization patterns depended on both yeast strain and nitrogen availability. For low-nitrogen fermentation, three strains did not fully degrade the fructose.

This study validates phenotypic and metabolic diversity among commercial wine yeasts and contributes new findings on the relationship between nitrogen availability, yeast cell growth and sugar utilization. We suggest that measuring nitrogen during the stationary growth phase is important because yeast cells fermentative activity is not exclusively related to population size, as previously assumed, but it is also related to the quantity of nitrogen consumed during this growth phase.

## Introduction

In most wine**-**producing countries, inoculating grape**-**juice with active**-**dried selected yeasts is a routine practice that yields reliable fermentations and reproducible product as well as predictable sensorial properties and quality. Modern winemaking is based on a monoculture inoculation technology that began in Germany with Müller**-**Thurgau in 1896 and was widely established in the second half of the twentieth century. Alternative yeast species combined with inoculation strategies yield wines with very different chemical and flavor profiles, such as a greater flavor complexity and diversity as well as enhanced mouth feel and flavor persistence (Ugliano and Henschke [2009]). However, in certain types of wines, the yeast should minimally impact the varietal aroma derived from the grapes. The commercial yeast strains are natural isolates from vineyards or derived from breeding programs and are selected based on certain phenotypic characteristics, including tolerance to temperature variation, ethanol and SO_2_, low foaming production, completion of alcoholic fermentation to dryness (residual sugars <2 g l^−1^), no off**-**flavor production, including sulfides, and production of interesting aroma compounds (Boulton et al. [1996]). During alcoholic fermentation, yeasts encounter a combination of several stress conditions. Yeasts are exposed to a number of factors due to the composition of grape-juice, such as high sugar levels, variable nitrogen concentration, low pH and potential inhibitors, including SO_2_ and temperature variation. In addition, there are several stress conditions established by the yeast, including increased ethanol and inhibitor production. Yeast performance during alcoholic fermentation greatly depends on its ability to adjust to continuous environmental changes. The active dry yeast performance, including fermentation capacity and flavor release, greatly depends on the genetic background and fermentation conditions (Pretorius [2000]). Estimates indicate that the market includes over 200 available yeast strains that are used globally for winemaking, and these are primarily *Saccharomyces cerevisiae* strains. In recent years, large-scale systematic studies on the genetic and phenotypic diversity of *S. cerevisiae* populations have emerged (Kvitek et al. [2008]; Liti et al. [2009]; Camarasa et al. [2011], Dunn et al. [2012]; Richter et al. [2013]). These studies used a high-throughput stress resistance analysis or adaptation to diverse environments (carbon and nitrogen sources, presence of toxins and nutrient limitations) to characterize the phenotypes of the yeast strains of interest, such as strains related to wine-making or fermentation of other alcoholic beverages (Camarasa et al. [2011]; Nikolaou et al. [2006]; Zuzuarregui and del Olmo [2004]; Kvitek et al. [2008]). In this work, we aimed to (i) study the phenotypic diversity of a small subset of commercial wine yeasts; (ii) assess their nitrogen requirements for growth and fermentation; and (iii) analyze their growth kinetics and metabolic traits. This study provides additional potential criteria for inclusion in a yeast selection program.

## Material and methods

### Yeasts and maintenance conditions

Twenty *S. cerevisiae* strains, all wine strains except K7 which is a sake yeast strain, from geographically diverse environments (Table 1) were characterized in this study. The strains were sourced from different yeast culture collections, such as the Oenology Culture Collection of the Department of Viticulture and Oenology, University of California, Davis, USA and the National Research Institute of Brewing (NRIB), Japan; the additional cultures were acquired from the market.

**Table 1:** Commercial yeasts used in this study and respective origin countries and suppliers

**Yeast strain**	**Geographical origin**	**Supplier**
T73	Spain	Lalvin
EC1118	France	Lalvin
FERMIVIN	France	DSM
ZYMAFLORE VL1	France	Laffort
QA23	Portugal	Lalvin
CEG	Germany	UVAFERM
VIN 13	South Africa	Anchor
NT116	South Africa	Anchor
BM45	Italy	UVAFERM
BRL97	Italy	UVAFERM
FERMICRU XL	Chile	DSM
FERMICRU LVCB	Chile	DSM
XLD	Chile	Yeasts Collection DBVPG, Chile
UCD522	USA	Maurivin
UCD595	USA	UC Davis Collection, California, USA
UCD505	USA	UC Davis Collection, California, USA
AWRI796	Australia	Maurivin
AWRIR2	Australia	Maurivin
W3	Japan	National Research Institute of Brewing (NRIB), Higashiroshima, Japan
K7	Japan	National Research Institute of Brewing (NRIB), Higashiroshima, Japan

The strains were collected aseptically from active dried commercial preparations, re**-**hydrated in sterile water with 50 g l^−1^ of glucose in accordance with the manufacturer’s instructions (37°C, for 30 min) and inoculated into yeast peptone dextrose medium (YPD), which included, per liter, 20 g glucose, 10 g peptone and 5 g yeast extract. The cultures were then streaked onto Wallenstein agar plates (WL) and grown at 30°C to determine the purity. Multiple representative colonies were inoculated into YPD broth and were starter cultures for the stress resistance assays. Pure cultures were routinely maintained at 4°C on YPD slants, and the stocks were stored at −80°C with glycerol at a final concentration of 40% (v/v).

### *S. cerevisiae* physiological and metabolic characterization

#### Stress resistance assays

To systematically analyze the particular stresses that affect yeast during wine production stages, each yeast strain was grown under different conditions that represent six environments to induce various physiological responses. The cells were grown in YPD medium until the mid-exponential growth phase (OD_600nm_ = 1), and three 10-fold serial dilutions were generated. For each strain, the four culture suspensions were spotted onto YPD agar plates with the appropriate stress and onto YPD agar plates without a stress agent, as a control, followed by incubation for 2–3 days, at 30°C.

#### Temperature variation

The tolerance to temperature variations was evaluated by spotting 5 μl of the dilutions from each yeast culture onto YPD agar plates after incubation at 15, 37 or 40°C, for 1–3 days.

#### Osmotic and oxidative shocks

The osmotic and oxidative shock tolerances were evaluated by spotting 5 μl of the dilutions from each yeast culture onto YPD agar plates with 1, 1.5 or 2 M of NaCl (from a 5 M stock solution) or 1.5 or 2.5 mM of H_2_O_2_ (from a 10 mM stock solution), respectively.

#### Ethanol resistance

Ethanol tolerance was evaluated by spotting 5 μl of the dilutions from each yeast culture onto YPD agar plates with different ethanol concentrations (10, 12.5, and 15% [vol/vol]).

#### Sulfur dioxide (SO_2_) resistance

The sulfur dioxide resistance was evaluated based on the cell growth under different SO_2_ concentrations (2, 4 and 6 mM) through adding 5 ml of a stock solution (30, 60 and 90 mM) to the YPD medium at pH 3.5.

#### Acetic acid resistance

To evaluate the capacity of yeast to grow in the presence of acetic acid, 5 μl of the dilutions from each yeast culture were spotted onto a YPD medium containing 90, 110 or 150 mM acetic acid from a 5 M stock solution at pH 4.5.

Each yeast strain was tested for each stress at least twice at the aforementioned 2–3 doses for most stresses on three different occasions. Final resistance scores for the initial suspension (OD_600nm_ = 1) and the 3 serial 10**-**fold dilutions were summed, and the resulting scores were then averaged for the replicates and the stress doses, providing a single score ranging from 0 (no growth) to 8 (active growth) for each strain and each stress condition.

### Identifying 5, 5′, 5″-trifluor-D, L-leucine (TFL)- and/or cerulenin-resistant strains

To detect strains that produced certain flavor compounds, the strains were tested for the capacity to grow in the presence of TFL and/or cerulenin. The screens were conducted using agar plates and minimal medium (YNB) supplemented with glucose (2% [wt/vol]) and TFL (1 mM) (Oliveira et al. [2008]) or cerulenin (6 μM) (Franco**-**Duarte et al. [2009]) followed by incubation at 30°C for 3 days. For the remaining resistance assays, the final resistance scores were calculated using the above-described method.

### Evaluation of sulfite reductase activity

To examine the capacity to produce H_2_S, the sulfite reductase activity was estimated using a commercially available bismuth-containing agar (Bacto Biggy agar, Difco, Sparks, MD, USA). The culture media was prepared according to the manufacturer’s instructions. After 48 hours at 30°C, H_2_S formation was evaluated based on the varying colony color intensities, which turn light brown to black or remain white depending on the level of production (Mendes-Ferreira et al. [2002]). The yeast growth scores were based on the colony color, which ranged from white (scored 0) through brown to near**-**black (scored 8).

### Inoculum preparation and fermentation conditions

Based on the results, eight out of twenty strains were selected for fermentation experiments under two nitrogen regimes. Each yeast strain was rehydrated in accordance with the manufacturer’s instructions (in water with 50 g l^−1^ of glucose, at 37°C, for 30 min) and inoculated in synthetic grape-juice media (GJM) at an initial cell count of 10^6^ CFU/ml. The GJM, similar in the composition to typical grape juice, was formulated as previously described by Henschke and Jiranek ([1993]), with minor modifications. An equimolecular mixture of glucose and fructose (100:100 g l^−1^) was used as carbon and energy source, and the yeast assimilable nitrogen (YAN) was supplied as di**-**ammonium phosphate (DAP) for the sole nitrogen source to facilitate monitor the nitrogen consumption profile. To evaluate the yeast nitrogen demands for growth and fermentation, two initial nitrogen concentrations were used: 67 mg l^−1^, which yields a nitrogen-limiting condition that produces sluggish fermentation (Mendes**-**Ferreira et al. [2009]), or 10**-**fold higher (670 mg l^−1^), under which nitrogen is in excess. The pH was adjusted to 3.7 with NaOH prior to sterilization. The fermentation experiments were conducted in 250 ml**-**flasks filled to 2/3 of their volume and fitted with a side-arm port sealed and a rubber septum for anaerobic sampling; the conditions were maintained at 20°C in an orbital shaker at 120 rpm. The experiments were monitored daily for weight loss, which estimates CO_2_ production. Aseptic samples were collected daily to assess fermentation and growth parameters using a syringe**-**type system, in which the fermentation gasses escaped through a glass tube connected to a two**-**way valve through Teflon tubing connected to a fermentation lock and an H_2_S trap, as previously described (Mendes**-**Ferreira et al. [2009]). Every 24 h, the flasks were removed from the shaker, weighed, disconnected from the trap, and connected to a new trap. Prior to sampling, the flasks were stirred to ensure homogeneity. The experiments proceeded until no further weight was lost, and completion was confirmed by stable sugar levels (< 2 g l^−1^) using Clinitest tablets (Roche, Amadora, Portugal). The cells were collected at 5 different time-points: 12, 24, 36 and 96 hours after inoculation and at the end of the alcoholic fermentation. The supernatants were stored at −20°C for further analysis. The maximum fermentation rate was determined using the slope from the linear dependence of the steepest decline in weight (g) at the corresponding time points (h). Each set of fermentation experiments was repeated at least three times, and the reported data are mean values of these replicated experiments.

### Determination of yeast growth and viability during fermentation

The yeast growth was followed by periodic optical density measurement (640 nm) of the appropriately diluted culture samples in a Shimadzu UV**-**2101 spectrophotometer (Shimadzu, Kyoto, Japan) and by counting the colony-forming units in YPD plates after incubation at 30°C, for 48 h. Further, the yeast cell biomass was determined using 2 × 50 and 3×15 ml samples centrifuged in pre**-**weighed tubes for 10 min at 2300 × g, washed twice with sterile deionized water, dried for 24 h at 100°C, and stored in a desiccator before weighing.

To evaluate the viability of nitrogen-starved cells, a sample from each low-nitrogen fermentation was spread onto yeast carbon base (YCB, Difco) agar plates with 0.73 mg N l^−1^ as amino acids: histidine.HCl (1 mg l^−1^), methionine (2 mg l^−1^) and tryptophan (2 mg l^−1^). YCB was used to test the ability of yeasts to assimilate nitrogen by adding diverse nitrogen sources. The histidine, methionine and tryptophan concentrations were reduced to 10% of their original concentration in the yeast nitrogen base. This experiment was expected to detect viable cells in solid media without a nitrogen source.

### Analytical determinations

#### Nitrogen determination

The ammonium concentration was determined during fermentation using a continuous**-**flow analysis system as previously described (Mendes**-**Ferreira et al. [2004]). The maximum nitrogen consumption rate was determined by the slope from the linear dependence of the steepest decline in nitrogen concentration (mg) at the corresponding time points (h). The nitrogen consumption rates during the stationary growth phase under the high nitrogen regime were calculated using the slope from the linear dependence between nitrogen consumed (mg) and the respective duration (h).

#### H_2_S determination

The quantity of H_2_S released by the cultures was determined colorimetrically following selective collection of fermentation gases using a modified fermentation lock and sulfide**-**trapping system, as described previously (Mendes-Ferreira et al. [2009]). The sulfide concentration was calculated using a calibration curve established from known sulfide quantities (0–12 μg) and standard methods (Acree et al. [1971]; Rees et al. [1971]).

#### Fermentation metabolites determination

The glucose and fructose consumed, ethanol converted, and glycerol, succinic and acetic acids produced were analyzed for each sample throughout fermentation using high**-**performance liquid chromatography (HPLC) and a liquid chromatographic system equipped with the ion exclusion, cation exchange column Aminex HPX**-**87H (Bio-Rad Laboratories, Hercules, CA, USA) and a refractive index detector. The column was eluted using sulfuric acid (0.013 N) at 62°C and a 0.6 ml min^−1^ flow rate. The samples were filtered through a membrane (Millipore, 0.22 μm pore size) before injecting 6 μl. The components were identified through their relative retention times compared with the respective standards, using the EZChrom Elite (vers. 3.x) Software (Agilent Technologies, Inc., Santa Clara, CA USA).

### Statistical analysis

The strains were clustered based on the phenotype scores obtained, using the Pearson correlation and UPGMA clustering (Spotfire 4.5 – TIBCO Software Inc., 2007–2012, Somerville, Massachusetts, USA). Factorial design experiments were used to analyze the influence of the two nitrogen concentrations and eight yeast strains on metabolic activity. The effects of nitrogen concentration on each strain’s fermentation activity were examined through a two**-**way analysis of variance (ANOVA) and principal component analysis (PCA) using SAS JMP 7.0 (2007, Cary, NC, USA). For paired comparisons, the Tukey HSD (honestly significant difference) test was used. *p* < 0.05 was considered statistically significant.

## Results

### Phenotypic characterization

**Figure 1:**
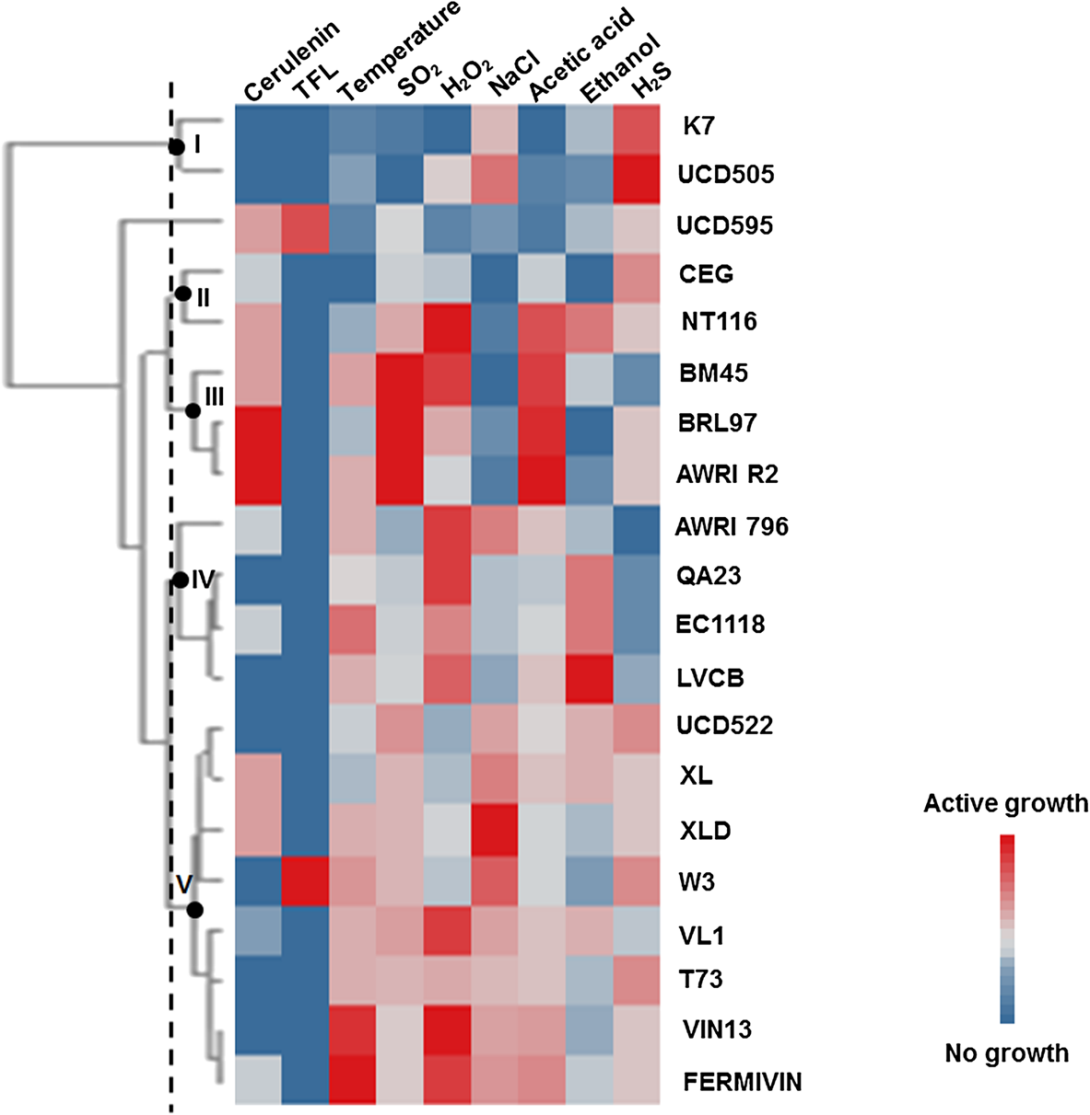
**Phenotypic variation in the 20 commercial yeast strains.** The strains were grown at least in duplicate on solid agar plates with 1–3 doses of each environmental variable, as described in Material and Methods. Each row on the plot represents a different strain, and each column indicates a particular environment. The colored boxes represent the average growth score of each strain in each environment, as in the legend shown at the lower right. For H_2_S, the color gradient indicates the color intensity of the colonies on the BIGGY agar, low or absent (blue) and high (red).

Considering the objectives of this study, the strains were characterized for relevant alcoholic fermentation features, such as tolerance to temperature variation, presence of oxidizing agents and osmotic/ionic stress as well as the ability to produce certain volatile compounds. The phenotypic characteristics data are depicted in Table 2 and the Additional file 1: Table S1 as final scores for each strain and stress condition, ranging between 0 (no growth) and 8 (active growth). Additional file 1: Figure S1 shows examples of yeast growth under the different stress conditions. The strains and conditions were organized through hierarchical clustering using the Pearson correlation and UPGMA clustering, which were used to separate the isolates into distinct groups, as shown in Figure 1.

**Table 2:** Phenotypes mean scores and conditions used to cluster the commercial yeasts tested

**Yeast strain**	**Cerulenin**	**TFL**	**Temperature**	**SO**_**2**_	**H**_**2**_**O**_**2**_	**NaCl**	**Acetic acid**	**Ethanol**	**H**_**2**_**S**
K7	0.0	0.0	4.0	0.0	6.0	3.7	1.0	1.0	8.0
W3	0.0	2.0	6.3	5.7	5.3	4.0	3.0	1.2	6.4
UCD595	1.0	1.5	3.3	5.0	3.3	0.8	0.8	1.5	5.6
UCD505	0.0	0.0	3.3	0.7	2.5	2.7	0.5	1.5	7.2
XLD	1.0	0.0	6.0	5.7	5.8	5.0	3.0	1.5	5.6
T73	0.0	0.0	6.0	5.7	6.3	2.7	3.3	1.5	6.4
AWRI R2	2.0	0.0	6.0	8.0	5.8	0.3	4.8	1.0	5.6
LVCB	0.0	0.0	6.0	4.8	6.8	1.2	3.3	4.2	4.0
QA23	0.0	0.0	5.5	4.3	7.0	1.7	2.7	3.0	3.2
BRL97	2.0	0.0	4.7	8.0	6.3	0.7	4.7	0.7	5.6
EC1118	0.5	0.0	6.8	4.7	6.5	1.7	3.0	3.0	3.2
CEG	0.5	0.0	2.8	4.7	5.3	0.0	2.8	0.7	6.4
FERMIVIN	0.5	0.0	8.0	5.3	7.0	3.2	3.8	1.7	5.6
VIN13	0.0	0.0	7.7	5.3	7.3	3.0	3.7	1.3	5.6
BM45	1.0	0.0	6.2	8.0	7.0	0.0	4.5	1.7	3.2
NT116	1.0	0.0	4.3	5.8	7.3	0.3	4.3	3.0	5.6
XL	1.0	0.0	4.7	5.7	5.0	3.5	3.3	2.3	5.6
UCD522	0.0	0.0	5.2	6.2	4.5	3.0	3.2	2.3	6.4
VL1	0.3	0.0	6.0	6.0	7.0	3.0	3.3	2.3	4.8
AWRI796	0.5	0.0	6.0	3.0	7.0	3.5	3.3	1.5	2.4

### Yeast response to different stress conditions

The data herein demonstrate that temperature largely affects the growth of the yeast strains; each displayed active growth with a maximum score at 37°C, and 70% of the strains grew at 40°C, but eight out of 20 did not grow at 15°C. The strains K7, UCD505, UCD595, CEG and NT116 did not grow at 40°C but did grow at 15°C. The strains K7 and UCD505 diverged due to their low tolerance for most stress agents (acetic acid, SO_2_ and ethanol), particularly UCD505. The yeasts in cluster II (CEG and NT116) diverged from the cluster III yeasts (BRL97, BM45 and AWRI2) because the latter exhibited a strong resistance to acetic acid, SO_2_, and H_2_O_2_. Cluster V is enriched with the yeasts that were more resistant to temperature variations and H_2_O_2_ but had an average resistance to NaCl and acetic acid. Seventy-five percent of the yeast strains showed tolerance for the temperature range from 15 to 40°C, whereas 90% tolerated an acetic acid concentration up to 110 mM. Two strains, AWRI R2 and BM45, were particularly resistant to ethanol and acetic acid environmental stresses. Ethanol and sulfur dioxide resistance also varied among the strains. However, for the concentrations used, the strains were more sensitive to ethanol than sulfite; 85% of the strains did not grow with 12.5% ethanol, whereas only one, LVCB, tolerated 15% ethanol. For sulfur dioxide, 50% of the strains tolerated this antiseptic over the range 2 to 6 mM, but three, AWRI R2, BRL97 and BM45, reached maximum growth score with 6 mM of SO_2_. In sum, the presence of NaCl, H_2_O_2_ and acetic acid best discriminated the strains compared with the remaining stress conditions. In contrast, TFL was the least discriminating agent, because only 2 strains, UCD595 and W3, were resistant.

### The ability of yeasts to produce volatile compounds

TFL and cerulenin resistance were examined to identify the yeasts’ ability to produce flavoring compounds. The experiments were performed on agar plates with cerulenin and TFL added separately and scored for no growth/active growth after incubation for 3 days. The yeasts showed generally greater resistance to cerulenin compared with TFL. Sensitivity to TFL was a feature of most strains, except UCD595 and W3. Conversely, 60% of the strains presented resistance to cerulenin, even with low growth scores. Only one strain, UCD595, presented resistance to both cerulenin and TFL.

For H_2_S, the strains K7 and UCD505 displayed the highest sulfite reductase activities on Biggy agar (cluster I). The remaining strains exhibited variable colony color intensities, which indicate different abilities to produce H_2_S. Based on the color intensity of the colonies, the yeasts screened herein were classified as high (K7 and UCD595), medium (CEG, UCD522, W3, T73), low (QA23, EC1118, BM45) and no sulfide producers (AWRI796). The remaining strains displayed a low to moderate ability to produce sulfide.

### Yeast growth kinetics and fermentation profiles

**Figure 2:**
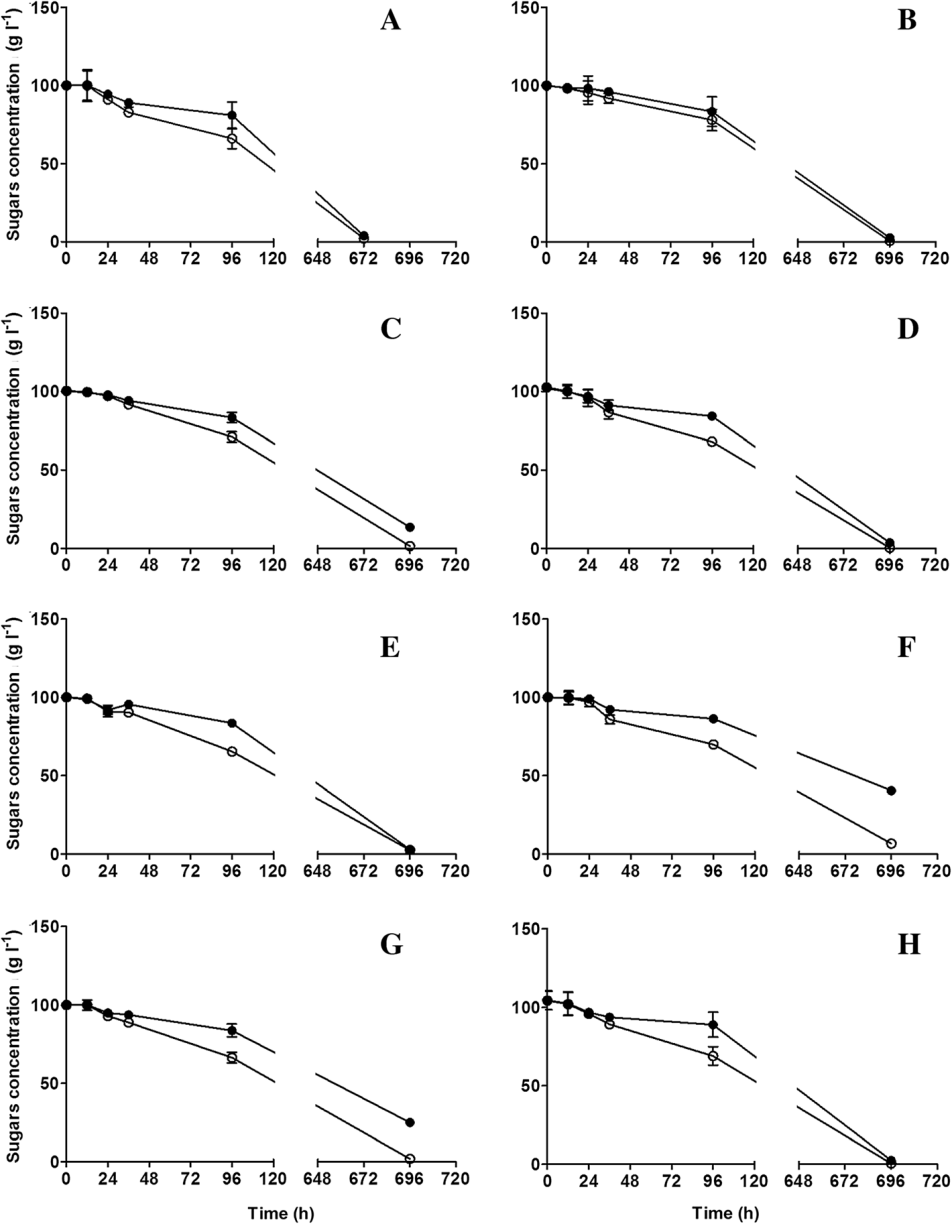
**Glucose (○) and fructose (●) consumption profiles compared for the 8 yeast strains QA23 (A), VL1 (B), CEG (C), BRL97 (D), XL (E), T73 (F), NT116 (G) and UCD522 (H) in the media supplemented with low nitrogen (67 mg l**^**−1**^**).**

**Figure 3:**
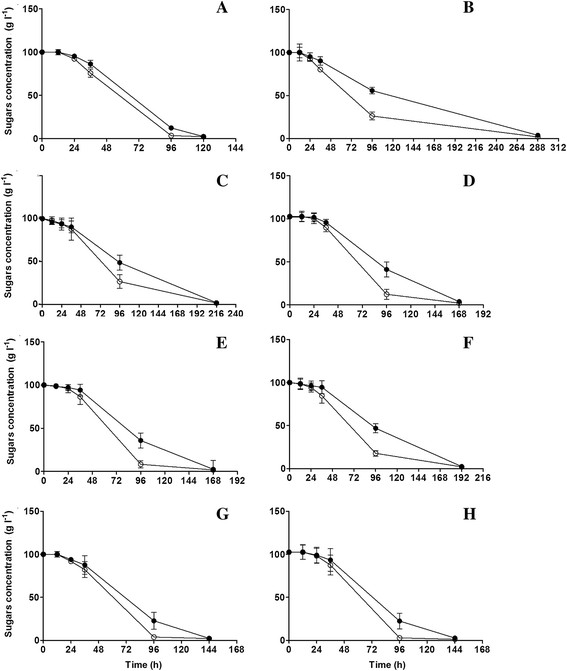
**Glucose (○) and fructose (●) consumption profiles compared for the 8 yeast strains QA23 (A), VL1 (B), CEG (C), BRL97 (D), XL (E), T73 (F), NT116 (G) and UCD522 (H) in the media supplemented with high nitrogen (670 mg l**^**−1**^**).**

Eight representative strains from the distinct phenotypic characterization groups regarded as strains with high (VL1, BRL97, T73, NT116), medium (QA23, UCD522, XL) and low resistance to stress (CEG) and with different abilities to produce H_2_S were used for fermentation experiments under two nitrogen regimes. The growth and fermentation parameters are in Table 3. Accordingly, vigorous fermentation was associated with high nitrogen levels and a high cell biomass. High nitrogen levels enhanced yeast growth and yielded higher fermentation rates with lower durations. With excess of nitrogen, the fermentation rates were much higher and the differences among the strains were great, whereas low nitrogen fermentations yielded relatively similar results among strains tested. Under the former conditions, 120 to 288 h were required for complete dryness depending on the yeast strain, whereas under the latter conditions, only five strains, QA23, UCD522, BRL97, VL1, and XL completed alcoholic fermentation after 672 to 696 h. The strains NT116, T73 and CEG did not fully degrade the sugars under low nitrogen. As the fermentation length per se does not fully define the performance of each individual strain, the pattern of sugars utilization was analyzed to better discriminate the yeast strains and nitrogen effects (Figures 2 and 3). Although fermentations started with equal quantities of glucose and fructose, the simultaneous but slower fructose use compared with glucose, yielded differences between their respective concentrations and the fermentation length depended on fructose use. Discrepancies in sugar consumption under high nitrogen levels were clearer for certain yeasts, such as VL1, T73, BRL97 and XL (Figure 3B, D**-**F). The strains QA23 and VL1 were, respectively, the fastest and slowest nitrogen-consumers (Figure 3A**-**B).

**Table 3:** **Overview of growth and fermentation parameters of the eight wine yeast strains grown in synthetic grape juice medium under low (67 mg l**^**−1**^**) and high nitrogen (670 mg l**^**−1**^**) concentrations**

**Strain**	**N level (mg l**^**−1**^**)**	**Specific growth rate μ (h**^**−1**^**)**	**Viable cells/ml**	**Final biomass (g l**^**−1**^**)**	**Maximum fermentation rate (g h**^**−1**^**)**	**TN**_**100%**_**(h)**	**Maximum nitrogen consumption rate (mg h**^**−1**^**)**	**N**_**EP**_**(mg)**	**N**_**SP**_**(mg)**	**N**_**SP**_**rate (mg h**^**−1**^**)**	**Total N consumed (mg)**
**T73**	**67**	0.185 ± 0.00^b,c,d,e,f^	3.28 E + 07 ± 1.39 E + 07^e,f^	2.6 ± 0.06^h,i^	0.03 ± 0.01^g^	26.11 ± 0.00^e^	4.0 ± 0.01^c,d,e^	_	_	_	
	**670**	0.196 ± 0.008^b^	1.02 E + 08 ± 0.03E + 08^d^	6.6 ± 0.02^b,c^	0.11 ± 0.00^e,f^	_	5.0 ± 0.09^b^	240.9 ± 4.69^b,c^	84.8 ± 14.56^d,e^	0.6 ± 0.10^d^	333.9 ± 16.01^c,d^
**UCD522**	**67**	0.144 ± 0.01^g^	4.95E + 07 ± 0.07 E + 07^e^	2.5 ± 0.01^h,i,j^	0.04 ± 0.01^g^	35.03 ± 0.00^b^	3.2 ± 0.31^e,f,g^	_	_	_	
	**670**	0.156 ± 0.005^d,e,f,g^	1.10 E + 08 ± 0.01 E + 08^c,d^	5.8 ± 0.07^d,e^	0.19 ± 0.00^a^	_	6.0 ± 0.09^a^	288.4 ± 4.13^a^	120.7 ± 29.97^c,d^	1.3 ± 0.31^b^	409.1 ± 29.28^a,b^
**BRL97**	**67**	0.152 ± 0.01^f,g^	3.56 E + 07 ± 0.02E + 07^e,f^	2.1 ± 0.04^k^	0.03 ± 0.01^g^	32.6 ± 0.00^c^	3.1 ± 0.26^f,g^	_	_	_	
	**670**	0.160 ± 0.011^d,e,f,g^	1.03 E + 08 ± 0.03 E + 08^d^	5.6 ± 0.13^e^	0.14 ± 0.00^c,d^	_	4.4 ± 0.33^b,c,d^	211.3 ± 15.68^c,d^	117.3 ± 10.33^d^	1.0 ± 0.09^b,c^	328.6 ± 6.09^d^
**NT116**	**67**	0.186 ± 0.01^b,c,d,e^	4.45 E + 07 ± 0.3 E + 07^e^	2.4 ± 0.03^i,j^	0.04 ± 0.01^g^	24 ± 0.00^f^	4.4 ± 0.30^b,c,d^	_	_	_	
	**670**	0.184 ± 0.005^b,c,d,e,f^	1.50 E + 08 ± 0.10 E + 08^a,b^	6.7 ± 0.07^b^	0.16 ± 0.01^b,c^	_	2.7 ± 0.33^g,h^	131.2 ± 15.98^e^	255.3 ± 22.75^a^	2.7 ± 0.24^a^	386.5 ± 6.88^b^
**QA23**	**67**	0.170 ± 0.00^c,d,e,f,g^	4.36 E + 07 ± 0.21 E + 07^e^	2.3 ± 0.03^j^	0.04 ± 0.01^g^	22.34 ± 0.00^g^	3.7 ± 0.07^d,e,f^	_	_	_	
	**670**	0.173 ± 0.007^c,d,e,f,g^	1.71 E + 08 ± 0.08E + 08^a^	5.9 ± 0.07^d^	0.17 ± 0.01^a,b^	_	5.0 ± 0.38^b^	239.8 ± 18.47^b^	177.5 ± 11.68^b^	2.5 ± 0.16^a^	417.3 ± 6.96^a^
**VL1**	**67**	0.210 ± 0.01^a,b^	4.87 E + 07 ± 0.35 E + 07^e^	2.6 ± 0.04^h^	0.04 ± 0.01^g^	24 ± 0.00^f^	4.8 ± 0.31^b,c^	_	_	_	
	**670**	0.220 ± 0.004^a^	1.15 E + 08 ± 0.21 E + 08^c,d^	6.4 ± 0.05^c^	0.08 ± 0.01^f^	_	2.1 ± 0.12^h^	100.9 ± 5.66^f^	166.5 ± 8.87^b^	0.7 ± 0.04^c,d^	267.4 ± 12.28^d^
**CEG**	**67**	0.153 ± 0.00^e,f,g^	1.49 E + 07 ± 0.35 E + 07^f^	1.7 ± 0.06^l^	0.04 ± 0.00^g^	44.06 ± 0.00^a^	2.1 ± 0.11^h^	_	_	_	
	**670**	0.146 ± 0.002^g^	5.73 E + 07 ± 0.09 E + 07^e^	5.3 ± 0.09^f^	0.13 ± 0.01^d,e^	_	2.9 ± 0.12^f,g,h^	282.7 ± 13.24^a^	55.3 ± 1.43^e^	0.5 ± 0.01^d^	338.1 ± 14.68^c,d^
**XL**	**67**	0.192 ± 0.02^a,b,c,d^	3.80 E + 07 ± 0.28 E + 07^e,f^	3.1 ± 0.12^g^	0.04 ± 0.01^g^	30.33 ± 0.00^d^	2.9 ± 0.13^f,g,h^	_	_	_	
	**670**	0.195 ± 0.026^a,b,c^	1.32 E + 08 ± 0.07E + 08^b,c^	7.5 ± 0.01^a^	0.14 ± 0.01^c,d^	_	4.2 ± 0.61^b,c,d^	202.8 ± 29.23^d^	153.4 ± 32.15^b,c^	1.3 ± 0.27^b^	356.3 ± 4.61^c^

Values in same column not connected by the same letter are significantly different, *p* < 0.05.

N_EP_ - Nitrogen consumed during exponential phase.

N_SP_ - Nitrogen consumed during stationary phase.

N_SP_ rate - Nitrogen consumption rate during stationary phase.

TN_100%_ - Time necessary to consume all nitrogen.

The yeast sugar consumption differed under low and high nitrogen. Sugar concentrations decreased slightly at the beginning of fermentation (0 to 2 days) (Figure 2A**-**H) through the fourth day; the sugar consumption rate was similar for each yeast strain but with lower decay curves than in the high nitrogen media. As shown in Figures 2 and 3, the area between the glucose and fructose consumption curves was clearly affected by both nitrogen availability and the yeast strain used. The fructose utilization kinetics influenced overall fermentation for the individual strains. The strains T73, CEG and NT116 yielded a larger area between the consumption curves for both sugars, which yielded an incomplete fermentation, likely due to an inability to entirely degrade the fructose in the medium.

The yeast cell viability (CFU’s) and final biomass was significantly higher (2 to 3**-**fold) under high nitrogen, but each strain exhibited equal specific growth rates, irrespective of the initial nitrogen concentration used (Table 3).

The yeast cells viability remained constant (either in YPD or YCB medium) under low nitrogen, and the yeast cells slowly continued to consume sugars for almost a month without extracellular nitrogen. As shown in Table 3, more nitrogen was used by the yeasts upon increasing the available nitrogen in the media; the nitrogen was fully consumed after 22 to 44 h (depending on the strain) in the media with low nitrogen. High nitrogen levels yielded different consumption rates during either the exponential or stationary growth phase. Both results demonstrate that nitrogen demands are highly strain-dependent and suggest that measuring nitrogen during the stationary growth phase is important. The quantity of nitrogen consumed during the stationary phase was positively correlated with a higher cell number and better fermentation competence, rather than the maximum nitrogen consumption rate. The strains with high fermentation activity, QA23, UCD522 and NT116, were high nitrogen-consumers with high nitrogen consumption rates, particularly during the stationary phase (Table 3). The strains NT116 and VL1 displayed high nitrogen consumption rates during low-nitrogen fermentations.

### The effects of nitrogen concentration and yeast strain on H_2_S release

H_2_S release showed a strain**-**dependent variable response to nitrogen availability; despite this contrasting behavior, certain strains, QA23, NT116, CEG and BRL97, produced negligible quantities of H_2_S under the two nitrogen regimes (Table 4). In contrast, the strains BRL97 and CEG showed a direct relationship between the initial levels of nitrogen in the media and the quantity of H_2_S released. The quantity of H_2_S released during alcoholic fermentation was positively correlated with the color intensity of the colonies on Biggy agar (Table 4 and Figure 1), particularly for the high and medium producers, UCD522, T73, XL and VL1.

**Table 4:** **Overview of the levels of metabolites produced by the eight wine yeast strains grown in synthetic grape juice medium under low (67 mg l**^**−1**^**) and high nitrogen (670 mg l**^**−1**^**) concentrations**

**Strain**	**N level (mg l**^**−1**^**)**	**Time to reach dryness (h)**	**Residual glucose (g l**^**−1**^**)**	**Residual fructose (g l**^**−1**^**)**	**Succinic acid (g l**^**−1**^**)**	**Glycerol (g l**^**−1**^**)**	**Acetic acid (g l**^**−1**^**)**	**Ethanol (% v/v)**	**Total H**_**2**_**S (μg l**^**−1**^**)**
**T73**	**67**	>696	6.73 ± 1.65^a^	40.48 ± 2.23^a^	1.91 ± 0.02^a,b,c^	6.74 ± 1.49^a,b^	0.32 ± 0.06^b,c,d^	8.95 ± 0.31^f^	214.4 ± 97.1^a^
	**670**	192	2.09 ± 0.29^b^	2.50 ± 0.13^d^	0.54 ± 0.03^e^	8.63 ± 0.29^a,b^	0.79 ± 0.07^a^	11.60 ± 0.46^a,b,c^	44.7 ± 22.7^b,c^
**UCD522**	**67**	672	0.47 ± 0.04^b^	2.42 ± 0.04^d^	2.00 ± 0.12^a,b^	7.24 ± 0.54^a,b^	0.22 ± 0.05^c,d^	12.00 ± 0.00^a,b^	201.7 ± 19.3^a^
	**670**	144	1.38 ± 0.32^b^	2.77 ± 0.89^d^	0.34 ± 0.03^e^	7.66 ± 0.34^a,b^	0.39 ± 0.01^b,c^	12.30 ± 0.00^a^	191.2 ± 14.3^a^
**BRL97**	**67**	696	0.51 ± 0.01^b^	3.52 ± 0.16^d^	1.68 ± 0.04^c,d^	6.27 ± 0.21^b^	0.44 ± 0.00^b^	11.80 ± 0.20^a,b^	4.4 ± 3.8^c^
	**670**	168	2.03 ± 0.35^b^	3.69 ± 0.09^d^	0.39 ± 0.00^e^	8.79 ± 0.94^a^	0.71 ± 0.01^a^	11.83 ± 0.21^a,b^	8.9 ± 6.7^c^
**NT116**	**67**	>696	1.85 ± 0.61^b^	24.94 ± 3.62^b^	1.76 ± 0.09^a,b,c^	7.18 ± 0.35^a,b^	0.32 ± 0.01^b,c,d^	10.2 ± 0.17^e^	5.6 ± 3.0^c^
	**670**	144	2.23 ± 0.63^b^	2.43 ± 0.36^c,d^	0.38 ± 0.04^e^	8.00 ± 0.30^a,b^	0.45 ± 0.01^b^	11.85 ± 0.35^a,b^	2.1 ± 1.7^c^
**QA23**	**67**	672	2.08 ± 1.43^b^	3.89 ± 0.50^d^	1.69 ± 0.04^b,c,d^	6.24 ± 0.16^b^	0.16 ± 0.03^d^	11.50 ± 0.14^a,b,c,d^	0.0 ± 0.0^c^
	**670**	120	2.31 ± 0.76^b^	2.38 ± 0.07^d^	0.33 ± 0.01^e^	8.47 ± 0.11^a,b^	0.43 ± 0.01^b^	11.90 ± 0.10^a,b^	0.0 ± 0.0^c^
**VL1**	**67**	696	0.62 ± 0.09^b^	2.66 ± 1.09^d^	1.94 ± 0.22^a,b,c^	6.34 ± 1.06^b^	0.34 ± 0.04^b,c,d^	11.38 ± 0.34^b,c,d^	45.1 ± 14.1^b,c^
	**670**	288	1.70 ± 0.22^b^	3.00 ± 0.31^d^	0.47 ± 0.02^e^	8.32 ± 0.42^a,b^	0.73 ± 0.04^a^	11.75 ± 0.35^a,b,c^	4.4 ± 0.5^c^
**CEG**	**67**	>696	1.49 ± 0.02^b^	13.57 ± 0.61^c^	1.46 ± 0.00^d^	7.05 ± 0.63^a,b^	0.33 ± 0.11^b,c,d^	10.87 ± 0.13^d,e^	0.6 ± 0.0^c^
	**670**	216	1.77 ± 0.03^b^	1.99 ± 0.07^d^	0.36 ± 0.04^e^	7.27 ± 0.13^a,b^	0.69 ± 0.02^a^	11.37 ± 0.21^b,c,d^	5.0 ± 7.0^c^
**XL**	**67**	696	2.57 ± 0.00^b^	2.94 ± 0.00^d^	2.02 ± 0.00^a^	7.81 ± 0.00^a,b^	0.24 ± 0.00^b,c,d^	11.38 ± 0.02^a,b,c,d^	127.6 ± 14.1^a,b^
	**670**	168	1.66 ± 0.15^b^	2.16 ± 0.14^d^	0.44 ± 0.05^e^	8.16 ± 0.07^a,b^	0.77 ± 0.09^a^	11.63 ± 0.4^a,b,c^	35.6 ± 23.7^b,c^

Values in same column not connected by the same letter are significantly different, *p* < 0.05.

### The effects of nitrogen concentration and yeast strain on fermentation metabolites

At the end of fermentation, we quantified several metabolites, such as ethanol, glycerol, succinic and acetic acids and the residual sugars (Table 4). We observed differences in the final ethanol concentration, particularly under low nitrogen for the T73, NT116 and CEG strains, due to incomplete sugar degradation. The yeast strains differed in regard to the succinic acid concentration formed under high or low nitrogen, but each strain was more productive during low-nitrogen fermentations. Under the latter condition, the quantity of succinic acid produced by the yeasts can be directly correlated with the final biomass (Tables 3 and 4). We evaluated the glycerol concentration produced by each strain individually and observed that, even without significant differences, high glycerol concentrations were produced during high-nitrogen fermentations. The exception was the yeast strain CEG, which produced almost the same glycerol concentration under both nitrogen regimes (Table 4). With high nitrogen levels, glycerol production positively correlated with the final yeast biomass (Tables 3 and 4). Further, more acetic acid was produced during high-nitrogen fermentations, being directly correlated with fermentation length.

**Figure 4:**
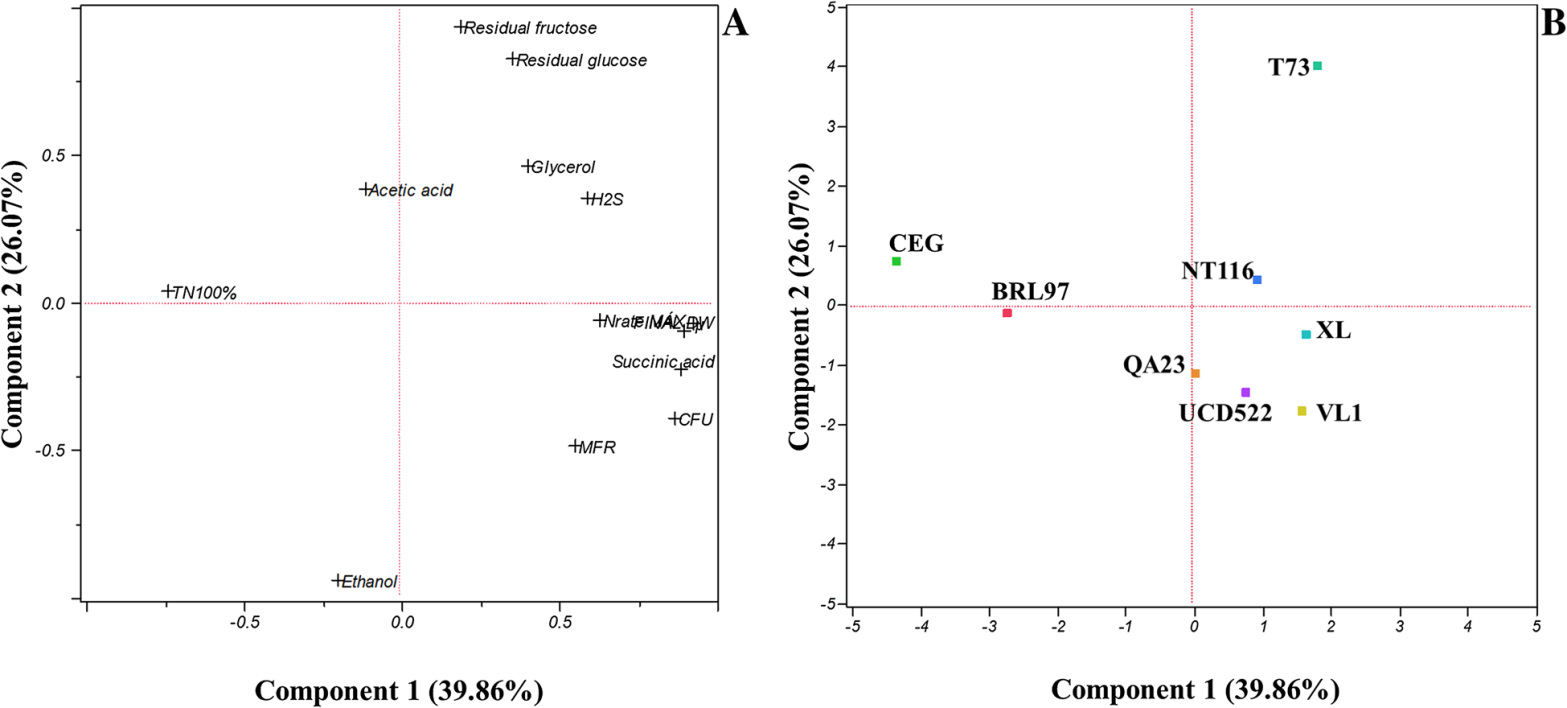
**Principal component analysis (PCA) plot using the mean values of the metabolite concentrations as well as growth and fermentation parameters of the 8 strains under a low nitrogen regime (67 mg l**^**−1**^**), the variable loadings (A) and scores (B) for the two first principal components.**

**Figure 5:**
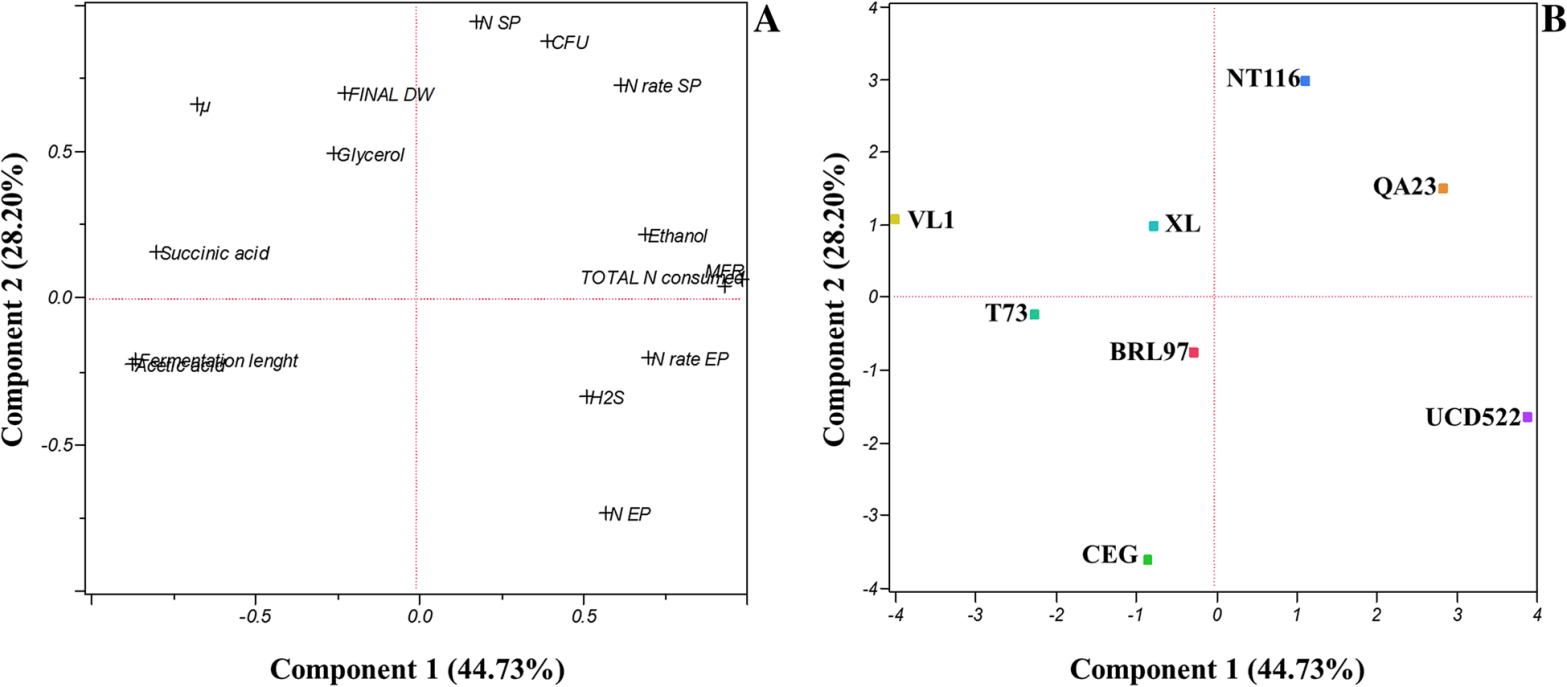
**Principal component analysis (PCA) plot using the mean values of metabolite concentrations and growth and fermentation parameters of the 8 strains under a high nitrogen regime (670 mg l**^**−1**^**), the variable loadings (A) and scores (B) for the two first principal components.**

For a general overview of the data, a principal component analysis (PCA) was performed using each strain and traits that characterized fermentations under the low**-** and high**-**nitrogen regimes (Figures 4 and 5). This approach was used to separate the strains based on growth parameters, nitrogen utilization and metabolites produced during fermentation. For fermentations under low nitrogen (Figure 4), PC1 and PC2 accounted for 42.8% and 25.4% of the variability, respectively. The strains were mainly discriminated by the time required to consume the available nitrogen (higher for CEG), nitrogen consumption rates and additional growth parameters. The center depicts the most vigorous/more rapidly fermenting yeast strains, QA23, UCD522 and NT116, and the upper right quadrant only indicates strain T73 due to the residual sugar levels. Clearly, under low nitrogen, certain direct relationships between the parameters analyzed can be established; the strains that exhibit higher nitrogen consumption rates have a higher final biomass, higher specific growth and maximum fermentation rates, and high succinate concentrations. Thus, at low nitrogen levels, the nitrogen consumption rate defines the cell biomass yield; XL, T73 and VL1; NT116, QA23 and UCD522; and BRL97 and CEG are high, medium and low biomass producers, respectively. For high-nitrogen fermentation (Figure 5), PCA explains 72.9% of the total variability; PC1 accounted for 44.7% and PC2 accounted for an additional 28.2%. The high maximum fermentation rates are mainly due to the nitrogen consumed and its consumption rate during the stationary phase, which yielded a high cell population and fermentation velocity. Glycerol formation directly correlated with yeast cell biomass. H_2_S liberation depended on the nitrogen consumption rate during the exponential growth phase. The strains were also discriminated by fermentation length; the more rapidly fermenting strains are located on the right quadrant (QA23, UCD522 and NT116), the medium in the center (BRL97 and XL), and the slowest on the left quadrant (CEG, T73 and VL1). Further, we observed a positive relationship between acetic acid formation and fermentation length.

## Discussion

The yeast stress response is important for survival and proper processing during fermentation. Indeed, it is a main research interest both for fundamental knowledge of the *S. cerevisiae* wine strains and for the practical application thereof (Bisson et al. [2007]). In this study, commercial yeast strains from different geographical origins were subjected to different environmental stresses that are similar to stresses encountered during alcoholic fermentation. Diversity among the strains was demonstrated through the unique phenotypic patterns of each strain, which is consistent with previous studies (Kvitek et al. [2008]; Mendes et al. [2013]). Overall, resistance to different NaCl, H_2_O_2_ and acetic acid concentrations was more strain**-**discriminating than the remaining stress conditions. *S. cerevisiae* yeast strains exhibit a higher natural predisposition for tolerating ethanol and sulfur dioxide (SO_2_), due to adaptation and natural selection, allowing them to predominate in many fermentation environments. The wine *S. cerevisiae* strains are more resistant to SO_2_, which has been a widely used preservative for centuries, due to higher *SSU1* gene expression, which encodes a plasma membrane sulfite pump that is required for efficient sulfite efflux and maintains sufficiently low sulfite levels to prevent toxicity (Park and Bakalinsky [2000]). Except for the sake yeast strain K7 (sake is sulfite-free), which was highly sensitive to the range of SO_2_ doses, all the strains tolerated the lowest concentration. Higher variability was observed among the yeast strains with the higher SO_2_ concentrations; the AWRI R2 and BM45 strains displayed the highest scores even with very high SO_2_ concentrations. For yeast tolerance to ethanol, we observed high diversity among the strains tested at 10%, which was diminished for the highest ethanol concentrations, wherein 80% of the strains did not grow. This is an intriguing result because a higher resistance phenotype was expected for the commercial wine strains. Additional studies have yielded similar results (Kvitek et al. [2008]). The low growth scores for the highest ethanol levels observed may be due to use of yeast cells in the exponential growth phase, wherein the cells are significantly more sensitive to environmental stress (Werner**-**Washburne et al. [1993]). An additional explanation may be that throughout alcoholic fermentation the yeast cells gradually adapt to the ethanol produced and increase their tolerance (Arroyo-López et al. [2010]). High resistance to oxidative stress is an advantage for wine yeast because hypoxic fermentation in a high**-**sugar medium induces yeast cells to produce ROS (Reactive Oxygen Species) (Mendes**-**Ferreira et al. [2010]; Landolfo et al. [2008]). Therefore, the strains that better manage oxidative stress from either yeast respiratory metabolism or the presence of H_2_O_2_ produced during alcoholic fermentation (Bisson et al. [2007]) will likely perform well during fermentation, such as for the QA23, FERMIVIN and VL1 strains herein. The high resistance to acetic acid demonstrated by the yeast strains AWRI R2, BRL97 and BM45, should be highlighted because this compound is known to affect yeast fermentative performance in winemaking. Additionally, it is important for yeast used in additional biotechnological processes, such as on bioethanol production, in which acetic acid is frequently the most dominant inhibitor in the plant**-**biomass hydrolysates (van Maris et al. [2006]).

From this screen, eight strains, UCD522, XL, FERMIVIN, VIN13, W3, XLD, T73 and VL1, should be highlighted because displayed interesting industrial features, such as a high resistance to temperature variation and oxidative shock as well as, simultaneously, an average resistance to acetic acid.

Despite the wide variation in growth scores under the different conditions tested, hierarchical clustering showed phenotypic similarity between certain strains irrespective of geographical origin. This phenomenon was described by Dunn et al. ([2012]), who observed similar pairs or groups of strains, but could not find clusters based on geographic origin using array**-**Comparative Genomic Hybridization (aCGH) for 83 different *S. cerevisiae* strains. These observations support the hypothesis that genomic diversity driven by mutation or DNA sequence alteration and environmental adaptation may impact *S. cerevisiae* strain diversity more than geographical origin.

Considering the major role of nitrogen availability in yeast growth and fermentative performance (Bisson [1999]; Salmon [1989]; Manginot et al. [1998]; Bell and Henschke [2005]), alcoholic fermentation trials experiments were conducted under two different nitrogen regimes. For that purpose, eight representative strains from distinct phenotypic clusters were used to evaluate the rates and profiles of nitrogen consumption as well as analyze its impact on fermentation kinetics and the metabolites produced. For each strain, nitrogen concentration affected the fermentation length, maximum fermentation rate and final biomass, not specific growth rates, as proposed in literature (Mendes**-**Ferreira et al. [2004,2009]; Gardner et al. [2002]; Varela et al. [2004]). Consistent with a recent study (Gutiérrez et al. [2012]) which demonstrated that the minimum nitrogen concentration required by yeasts to yield the μ_max_ is very low (5 to 18 mg N l^−1^), we did not detect significant differences in the yeast growth rates under the two nitrogen regimes examined herein. However, the highest fermentation rates were observed under high nitrogen conditions due to the higher final yeast cell biomass (Varela et al. [2004]; Albertin et al. [2011]). Nevertheless, for each nitrogen level and comparing yeast strains, the same correlation between yeast cell final biomass and better fermentative performance was not established. For instance, the VL1 strain final biomass was significantly higher than for QA23, although taking more 168 h to fully degrade the sugars. On the other hand, a positive correlation was found between nitrogen consumption during the stationary growth phase and fermentative activity. The strains with a shorter fermentation length, QA23, UCD522 and NT116, exhibited the highest nitrogen consumption rates, particularly during the stationary phase. These findings suggest that nitrogen is not necessarily directed towards biomass formation, but it might be used to generate high fermentative vigor, which is associated with fructose utilization (Berthels et al. [2004]). Fructose utilization and the time for its consumption impacted the fermentation length. The fructose consumption profile for the glucophilic wine yeast strains examined was dissimilar. The strains with a lower discrepancy between glucose and fructose consumption were assumed to perform better during fermentation (Berthels et al. [2004]), as herein for the yeast strains QA23, UCD522 and NT116.

Fermentation behavior under excess of nitrogen was not a predictor for fermentation efficiency under limited nitrogen. For instance, the strains T73 and CEG as well as, more importantly, the highly efficient strain NT116 did not complete alcoholic fermentation under low nitrogen conditions, which reinforces the notion that nitrogen availability during the stationary-growth phase is crucial for fructose consumption, at least for certain strains.

The wine yeast strains fermentation by-products differed quantitatively but not qualitatively; this metabolic diversity yields the individual wine organoleptic characteristics. Glycerol, succinic acid and acetic acid are the main secondary metabolites derived from sugars fermentation. In this study, the levels of glycerol in both nitrogen regimes did not differ significantly, most likely because glycerol is primarily formed due to an osmotic stress response (Nevoigt and Stahl [1997]), which occurred under both conditions. For acetic acid, although we observed quantitative differences among the strains, significantly higher levels were detected for high-nitrogen fermentations, as previously reported (Tromp [1984]; Mendes**-**Ferreira et al. [2009]; Barbosa et al. [2009]), being positively correlated with biomass production. This direct relationship has also been reported for succinic acid (Albers et al. [1996]). In contrast, herein succinic acid was formed at significantly higher levels under low nitrogen conditions, which is in agreement with Heerde and Radler ([1978]). Based on the results herein and considering that we did not observed significant differences in the levels of glycerol, the yeast cells likely use different mechanisms to balance the redox conditions under the two regimes. Under low nitrogen, the yeast cells likely produce succinic acid via the reductive branch of tricarboxylic acid cycle (TCA) to balance the NADH/NAD^+^ ratio (Heerde and Radler [1978]). Under high nitrogen the overproduction of NADPH produced by acetic acid formation is likely driven to yeast cell biomass and cellular redox balance is maintained without the need of succinic acid formation. Further investigation is required on the role succinic acid plays under varying fermentation conditions.

The cross-talk between phenotypic patterns, fermentation profiles and metabolic traits of commercial wine yeast strains allowed understanding their phenotypic relatedness. Moreover, this integrative study provides interesting data for inclusion in a yeast selection program and may be useful to the industry in designing better strategies to improve wine quality. This work contributes new findings on the relationships between nitrogen availability, yeast cell growth and sugars utilization.

## Competing interests

The authors have declared no conflict of interest.

## Additional file

## Supplementary Material

Additional file 1: Table S1. Raw phenotypes scores, conditions and stress doses used to characterize yeast strains. **Figure S1**: Growth variation of the 20 commercial yeast strains under control condition **(A)** and one dose of each stress agent tested: SO_2_ – 6 mM **(B)**; NaCl 1M (C); Temperature 40°C **(D)**; H_2_O_2_ – 2.5 mM **(E)**; Acetic acid 90 mM **(F)**; Cerulenin 6 μM **(G)** and TFL 1 mM **(H)**. Cells were spotted at concentrations of (from left to the right): 10^7^, 10^6^, 10^5^ and 10^4^ cells ml^-1^. Yeast strains were organized in three groups of four columns representative of the four cell suspensions. From the left to the right and top to the bottom, the first group of strains contains: K7, W3, UCD595, UCD505, XLD, T73, AWRI R2; the second: QA23, BRL97, EC1118, CEG, FERMIVIN, VIN13, BM45 and NT116; and the third group: XL, UCD522, VL1 and AWRI 796. Click here for file
