# Supplementary material for: Phenotypic and metabolic traits of commercial Saccharomyces cerevisiae yeasts
Source: AMB Express. 2014 May 10;4:39. doi: 10.1186/s13568-014-0039-6 (PMC4052691; doi:10.1186/s13568-014-0039-6)
Supplement: Additional file 1: Table S1. — Raw phenotypes scores, conditions and stress doses used to characterize yeast strains. Figure S1: Growth variation of the 20 commercial yeast strains under control condition (A) and one dose of each stress agent tested: SO2 – 6 mM (B); NaCl 1M (C); Temperature 40°C (D); H2O2 – 2.5 mM (E); Acetic acid 90 mM (F); Cerulenin 6 μM (G) and TFL 1 mM (H). Cells were spotted at concentrations of (from left to the right): 107, 106, 105 and 104 cells ml-1. Yeast strains were organized in three groups of four columns representative of the four cell suspensions. From the left to the right and top to the bottom, the first group of strains contains: K7, W3, UCD595, UCD505, XLD, T73, AWRI R2; the second: QA23, BRL97, EC1118, CEG, FERMIVIN, VIN13, BM45 and NT116; and the third group: XL, UCD522, VL1 and AWRI 796. [file s13568-014-0039-6-S1.docx]

Supporting information for**: Phenotypic and metabolic traits of commercial *Saccharomyces cerevisiae* yeasts**

Catarina Barbosa^1^, Patrícia Lage^1^, Alice Vilela^1^, Mendes-Faia A.^1^, Ana Mendes-Ferreira^1^*

^1^Institute for Biotechnology and Bioengeneering – Centre of Genomics and Biotechnology, Universidade de Trás-os-Montes e Alto Douro, Vila Real, Portugal.

*corresponding author: [anamf@utad.pt](mailto:anamf@utad.pt)

**Table S1** - Raw phenotypes scores, conditions and stress doses used to characterize yeast strains.
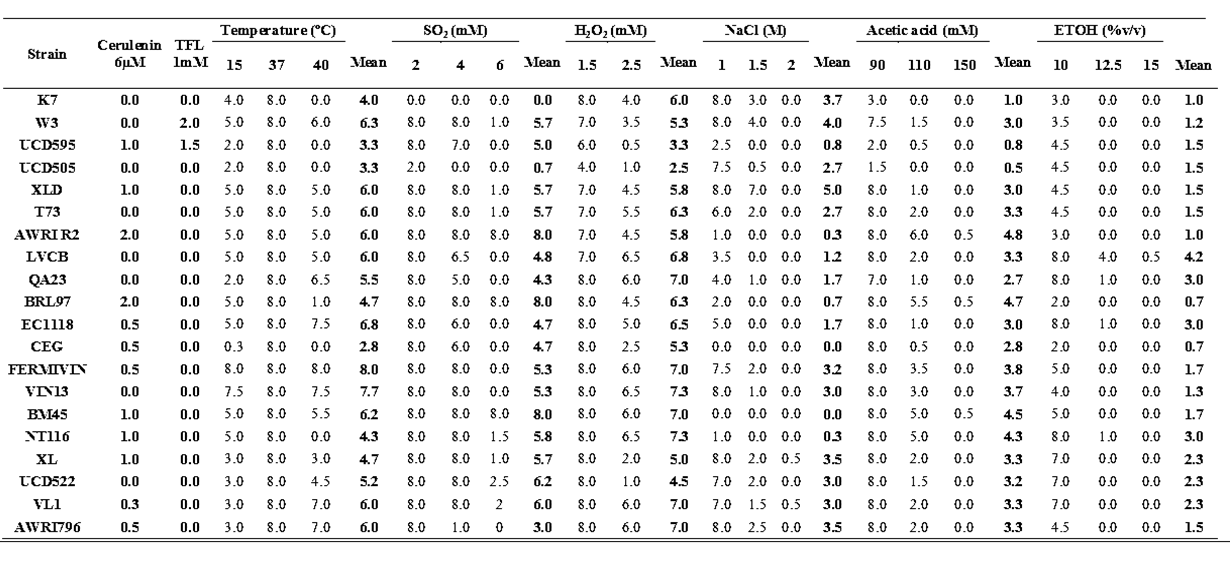


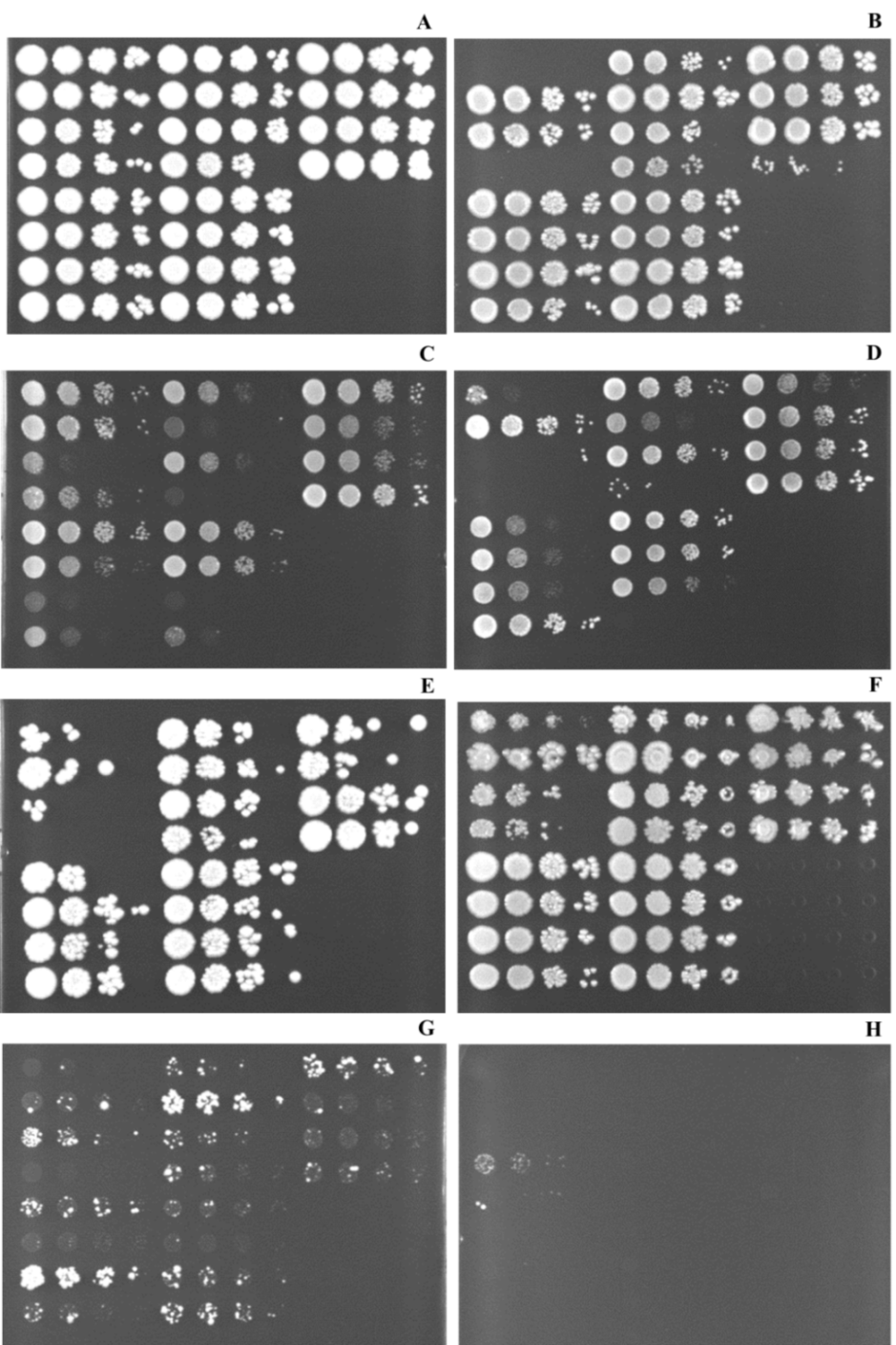


**Fig. S1** - Growth variation of the 20 commercial yeast strains under control condition (A) and one dose of each stress agent tested: SO_2_ – 6 mM (B); NaCl 1M (C); Temperature 40 ºC (D); H_2_O_2_ – 2.5 mM (E); Acetic acid 90 mM (F); Cerulenin 6 µM (G) and TFL 1 mM (H). Cells were spotted at concentrations of (from left to the right): 10^7^, 10^6^, 10^5^ and 10^4^ cells ml^-1^. Yeast strains were organized in three groups of four columns representative of the four cell suspensions. From the left to the right and top to the bottom, the first group of strains contains: K7, W3, UCD595, UCD505, XLD, T73, AWRI R2; the second: QA23, BRL97, EC1118, CEG, FERMIVIN, VIN13, BM45 and NT116; and the third group: XL, UCD522, VL1 and AWRI 796.
